# Uncovering potential biomarkers of endometriosis: transcriptomic and single-cell analysis

**DOI:** 10.3389/fmed.2025.1528434

**Published:** 2025-05-01

**Authors:** Yuqiu Liu, Guanwen Gao, Wei Tian, Qingfeng Lv, Degao Liu, Changzhong Li

**Affiliations:** ^1^Department of Ultrasound, Shandong Provincial Hospital Affiliated to Shandong First Medical University, Jinan, Shandong, China; ^2^Peking University Shenzhen Clinical Institute of Shantou University Medical College, Shenzhen, China; ^3^Center of Obstetrics and Gynecology, Peking University Shenzhen Hospital, Shenzhen, China; ^4^Shenzhen Key Laboratory on Technology for Early Diagnosis of Major Gynecologic Diseases, Shenzhen, China; ^5^Department of Obstetrics and Gynecology, Shandong Provincial Maternal and Child Health Care Hospital Affiliated to Qingdao University, Jinan, China; ^6^Department of Obstetrics and Gynecology, The Affiliated Taian City Central Hospital of Qingdao University, Taian, Shandong, China

**Keywords:** endometriosis, programmed cell death, mitochondria, single-cell, AIFM1, PDK4

## Abstract

**Background:**

The link between programmed cell death (PCD) and mitochondria has been documented in various diseases. However, its role in endometriosis (EMS) remains unexplored. This study aims to identify potential biomarkers in EMS associated with both PCD and mitochondrial functions.

**Methods:**

This analysis incorporates datasets related to EMS, PCD-related genes (PCD-RGs), and mitochondria-related genes (MRGs) sourced from public repositories. To uncover potential biomarkers, differential expression analysis, weighted gene co-expression network analysis (WGCNA), Boruta feature selection, expression validation, and diagnostic assessments were conducted. Functional analyses, immune infiltration profiling, and the construction of regulatory networks further elucidated the mechanisms through which these biomarkers may influence EMS. Finally, single-cell data were leveraged to examine the expression and functionality of these biomarkers at a granular level.

**Results:**

Apoptosis-inducing factor mitochondria-associated 1 (AIFM1) and pyruvate dehydrogenase kinase 4 (PDK4) were identified as potential biomarkers, with PDK4 upregulated and AIFM1 downregulated in EMS. Both genes demonstrated strong diagnostic potential. Enrichment analyses indicated their involvement in pathways associated with the cell cycle. Immune infiltration analyses revealed that AIFM1 had a significant positive correlation with resting dendritic cells and a negative correlation with M2 macrophages, whereas PDK4 was positively associated with M2 macrophages and inversely related to follicular helper T cells. Moreover, AIFM1 and PDK4 were regulated by 16 miRNAs (e.g., hsa-mir-16-5p) and 18 lncRNAs (e.g., LINC00294). Single-cell analysis further revealed dynamic expression trends of these potential biomarkers across cell differentiation stages, including gametocytes, monocytes, mesenchymal stem cells, and neutrophils.

**Conclusion:**

In this study, potential biomarkers (AIFM1 and PDK4) related to PCD and mitochondria were identified in EMS, offering valuable insights for the diagnosis and therapeutic strategies for the disease.

## 1 Introduction

Endometriosis (EMS) is characterized by the abnormal growth, infiltration, and recurrent bleeding of endometrial tissue (glands and stroma) outside the uterine cavity, leading to pain, infertility, and the formation of nodules or masses (1, 2). It is a prevalent condition among women of reproductive age (3). The development of EMS is influenced by various factors, including sex hormones, immune response, inflammation, and genetics, though its exact pathogenesis remains elusive. Current theories attempting to explain the origins of EMS include Sampson's Theory, immune dysregulation, Mullerianosis, vascular and lymphatic metastasis, the eutopic endometrium theory, and genetic and environmental contributions (4–7). Despite the prominence of these theories, none fully account for all cases (8), suggesting that EMS pathogenesis may be a multifactorial and stepwise process. Epidemiological studies have identified several risk factors for EMS, including family history, early menarche, short menstrual cycles, low fertility, obesity, chemical exposure, and prior abdominal surgery (9–12). A significant challenge in EMS management is diagnostic delay, which often leads to disease progression, complicates treatment, increases the likelihood of recurrence, and diminishes the patient's quality of life. Notably, no definitive cure exists for EMS, and current treatments primarily focus on symptom management. Early diagnosis is critical, yet no reliable biomarkers are available in peripheral blood or the endometrium for accurate diagnosis (13). Although elevated CA125 levels are often observed in patients with severe EMS, significant pelvic inflammation, or associated conditions like endometriotic cyst rupture or adenomyosis (14, 15), it is not a definitive diagnostic marker. Consequently, the identification of novel, effective biomarkers for early EMS diagnosis remains a pressing need to facilitate clinical management and improve treatment outcomes.

Programmed cell death (PCD) is a fundamental process in the development of multicellular organisms and plays a pivotal role in the pathogenesis of degenerative diseases. Key forms of PCD, including apoptosis, ferroptosis, and pyroptosis, involve mitochondrial participation (16). Mitochondria serve as critical regulators of PCD by acting as signal amplifiers. In response to both internal and external stimuli, mitochondria alter membrane permeability, release proteins such as cytochrome C, and initiate apoptotic pathways. Under normal conditions, mitochondrially produced reactive oxygen species (ROS) activate apoptosis; however, excessive ROS can damage mitochondrial integrity and accelerate apoptosis (17). Additionally, mitochondria interact with the endoplasmic reticulum and nucleus to propagate apoptotic signals (18). Mitochondrial dysfunction impairs cellular oxidative phosphorylation, leading to insufficient ATP production. To compensate, cells shift to anaerobic glycolysis, even in the presence of adequate oxygen, which produces lactate. This accumulation of lactic acid promotes cell migration, invasion, and angiogenesis, thus exacerbating the progression of EMS (19). Studies have underscored the pivotal role of PCD in the pathophysiology of EMS, suggesting potential therapeutic avenues. For instance, abnormal apoptosis in ectopic endometrial tissue is associated with disease progression and clinical manifestations (20). Additionally, spontaneous apoptosis is diminished in the endometrial glands of patients with EMS, facilitating immune evasion and promoting ectopic growth (21). Research has also shown that creatine confers resistance to ferroptosis in ectopic endometrial stromal cells by inhibiting prion protein, thus supporting EMS development (22). Furthermore, women with mild EMS exhibit a significantly higher number of dysfunctional mitochondria in their oocytes (23). Mitochondrial energy production and metabolism are impaired in EMS-affected tissues, potentially due to oxidative stress-induced mitochondrial DNA or membrane damage, metabolic shifts, or reduced availability of energy substrates. However, the combined impact of PCD and mitochondrial dysfunction on EMS pathogenesis remains unexplored. Thus, further investigation into the roles of mitochondria and PCD in EMS is essential.

Using a comprehensive bioinformatics approach, including machine learning techniques, this study identified biomarkers in EMS linked to both PCD and mitochondrial activity. Through expression validation and diagnostic performance analysis, the study not only assessed their potential clinical utility but also explored their expression dynamics. Integrated functional, immunological, and regulatory network analyses revealed the underlying mechanisms of these biomarkers in EMS. Additionally, single-cell level analysis provided new insights into their functional roles, offering a deeper understanding of EMS pathology and laying the groundwork for advancing diagnostic and therapeutic strategies.

## 2 Materials and methods

### 2.1 Data extraction

The GSE7305 dataset (platform GPL570) was downloaded from the Gene Expression Omnibus (GEO) database (https://www.ncbi.nlm.nih.gov/geo/) as a training set, comprising 10 EMS endometrial tissue samples (disease) and 10 control samples (normal). The GSE120103 dataset (platform GPL6480) was used as a validation set, including 18 EMS endometrial tissue samples and 18 control samples. Additionally, the GSE214411 single-cell dataset was downloaded from the GEO database, containing 6 EMS endometrial tissue samples and 7 control samples. A total of 1,136 mitochondria-related genes (MRGs) were obtained from the MitoCarta 3.0 database (http://www.broadinstitute.org/mitocarta) (24), and 1,548 programmed cell death-related genes (PCD-RGs) were retrieved from published literature (25).

### 2.2 Differential expression analysis

For the GSE7305 dataset, differential gene expression analysis was performed using the limma package (v3.51.0) (26), applying a threshold of an adjusted *p*-value < 0.05 and |log2(Fold change)FC| > 1 to filter differentially expressed genes (DEGs). Visualizations of DEGs were generated using volcano plots and heatmaps with the ggplot2 (v3.4.1) (27) and Complex Heatmap (v2.14.0) (28) packages.

### 2.3 Weighted gene co-expression network analysis (WGCNA)

In GSE7305, disease and normal samples were assigned as traits to identify the module most correlated with these traits using the WGCNA package (v1.71) (29). Hierarchical clustering based on Euclidean distance of gene expression levels was first conducted to identify and exclude outliers. A soft threshold with an *R*^2^ value >0.85 and close-to-zero connectivity was chosen as the optimal threshold. Using this, an unsigned network was constructed to categorize genes into modules (deepSplit = 2, minModuleSize = 30, mergeCutHeight = 0.25, numericLabels = FALSE, maxBlockSize = 100,000). Correlation analysis was subsequently performed to identify the key module most highly correlated with the traits, with its genes designated as key module genes.

### 2.4 Functional analysis

The intersection of DEGs, key module genes, MRGs, and PCD-RGs was determined using the ggVenn package (v1.7.3) (30), and the resulting intersecting genes were classified as candidate genes. Further functional analysis was conducted through Gene Ontology (GO) enrichment (adjusted *p*-value < 0.05) and Kyoto Encyclopedia of Genes and Genomes (KEGG) pathway analysis (*p*-value < 0.05) using the clusterProfiler package (v4.2.2) (31), to explore the biological functions and pathways associated with these candidate genes.

### 2.5 Identification of potential biomarkers

The Boruta package (v8.0.0) (32) was used for feature selection based on candidate genes to identify significant genes with confirmed importance. The expression of selected feature genes was then compared between the GSE7305 and GSE120103 datasets. Genes showing significant inter-group expression and consistent trends across both datasets were retained for subsequent analysis. Receiver operating characteristic (ROC) curves were plotted using the pROC package (v1.18.0) (33) in both datasets to evaluate the diagnostic performance of the genes. Genes with strong diagnostic performance (area under the curve [AUC] > 0.7) were defined as potential biomarkers.

### 2.6 Analysis of organizational expression

To further explore the expression patterns of potential biomarkers across human tissues, data from normal human tissues were analyzed using the Genotype-Tissue Expression (GTEx), Biological Gene Expression Profiling Source (BioGPS), and Serial Analysis of Gene Expression (SAGE) systems *via* the GeneCards database (https://www.genecards.org/).

### 2.7 Gene set enrichment analysis (GSEA)

GSEA was performed to investigate the key biological processes involved with the potential biomarkers. In the GSE7305 dataset, correlation coefficients between biomarkers and other genes were computed, and the genes were ranked in descending order. GSEA was then conducted using the c2.cp.kegg.v2023.1.Hs.symbols.gmt as the background gene set through the clusterProfiler package (v4.2.2) (34) (*p*-value < 0.05).

### 2.8 Immune infiltration analysis

Immune infiltration analysis was carried out based on the CIBERSORT algorithm to calculate enrichment scores for 22 immune cell types in all samples from the GSE7305 dataset. Wilcoxon tests were applied to compare enrichment scores between groups, excluding cell types with predominantly zero scores. Spearman correlation analysis (|cor| > 0.3, *p*-value < 0.05) was then performed to assess relationships between differentially enriched immune cells and potential biomarkers.

### 2.9 Regulatory analysis

To investigate upstream pathways associated with the potential biomarkers, the SPEED2 database (https://speed2.sys-bio.net/) was used to identify relevant pathways, with enrichment levels assessed using the Bates test to evaluate ranking changes. The pathways were ranked according to these changes, and gene rankings within each pathway were displayed. For molecular regulatory mechanisms, upstream miRNAs of the potential biomarkers were predicted using the ENCORI (http://starbase.sysu.edu.cn/index.php), miRcode (http://www.mircode.org/), and miRwalk (http://mirwalk.umm.uni-heidelberg.de/) databases. The intersection of predictions from all three databases was taken. Additionally, the ENCORI database was used to predict upstream lncRNAs for the identified miRNAs. A lncRNA-miRNA-mRNA (biomarker) regulatory network was constructed and visualized using Cytoscape software. The potential drugs related to the potential biomarkers were predicted using the Drug-Gene Interaction Database (DGIdb) (http://www.dgidb.org/), and the relationships between potential biomarkers and diseases were analyzed through the Comparative Toxicogenomics Database (CTD) (http://ctdbase.org/). Finally, the disease-biomarker-drug interaction network was visualized using Cytoscape software (v3.8.2) (35).

### 2.10 Single-cell analysis

The GSE214411 single-cell dataset was converted into a Seurat object using the Seurat package (v4.1.0) (36). Quality control was performed with criteria set as nFeature_RNA between 200 and 9,000, and percent.mt <25%. The data were normalized using the NormalizeData function, and highly variable genes were selected based on the relationship between mean and variance using the FindVariableFeatures function. Principal components (PCs) for subsequent analysis were determined *via* an Elbow Plot. Dimensionality reduction was performed using principal component analysis (PCA), followed by non-linear visualization with Uniform Manifold Approximation and Projection (UMAP) to identify cell clusters.

Specific genes with high expression in each cell cluster were identified using the FindAllMarkers function, with thresholds set at |log2FC| > 1 and an adjusted *p*-value < 0.05. Cell clusters were annotated using SingleR (37) for automatic annotation, along with marker genes from the Cell Marker database.

To differentiate between EMS and normal samples, cell communication analysis was conducted using the CellChat package (v0.0.7.900) (38) for each group separately. Functional enrichment analysis was performed using the ReactomeGSA package (v1.12.0) (39) to calculate pathway expression, followed by identification of differential expression pathways between EMS and normal samples, with the top 15 pathways visualized. Expression differences of biomarkers between EMS and normal groups across different cell types were also compared.

Pseudotime analysis was then performed using the Monocle package (v2.26.0) (40) to analyze the developmental trajectory of different cell types and assess the expression changes of potential biomarkers within these cells.

### 2.11 Statistical analysis

Data analysis was performed using R software (v4.1.0), with statistical significance evaluated using *t*-tests or Wilcoxon rank-sum tests, with a significance threshold set at *p* < 0.05.

## 3 Results

### 3.1 A total of 1,772 key module genes were identified in the GSE7305 dataset

**Figure 1:**
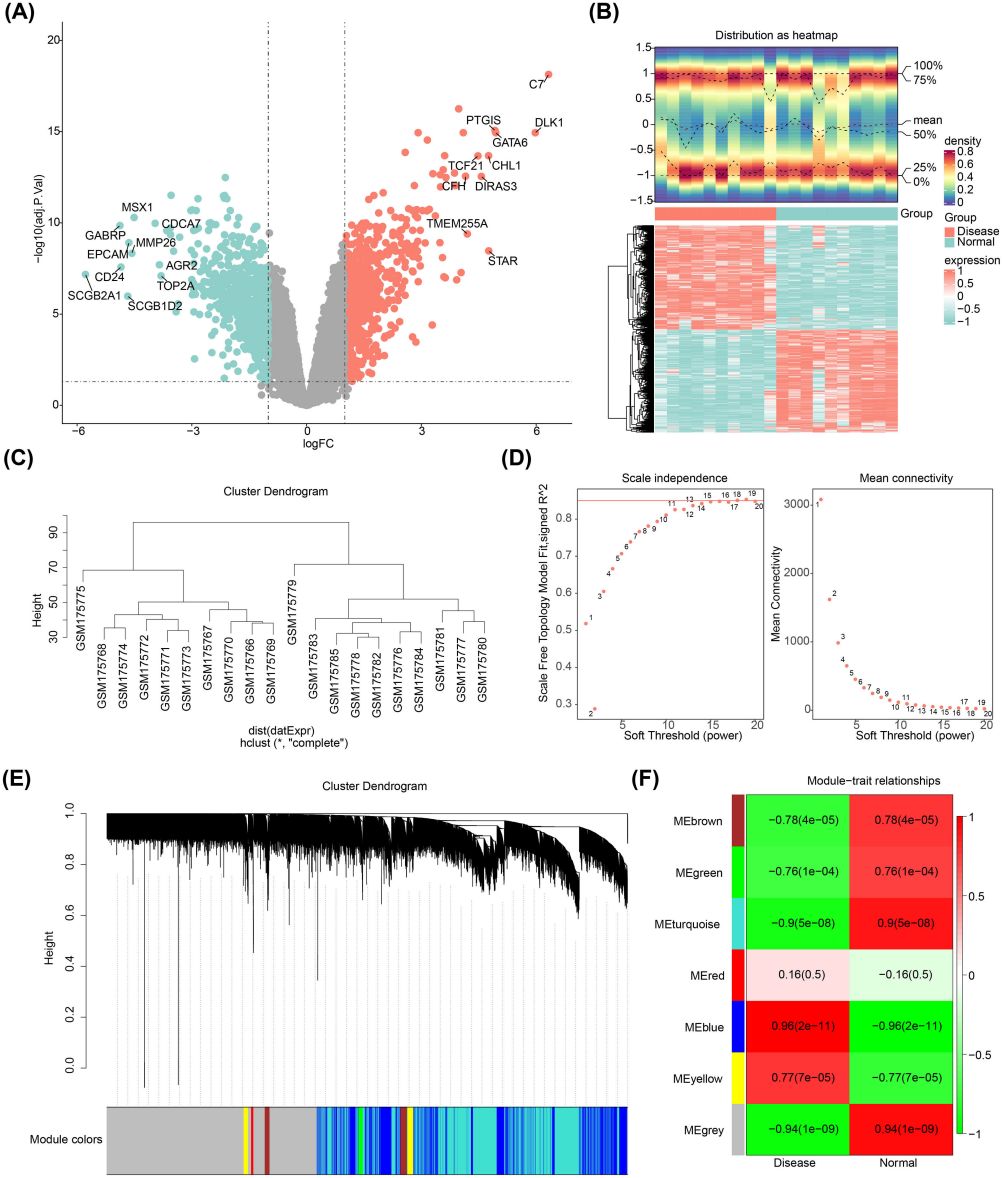
Differentially expressed genes in endometriosis. **(A)** Volcano plot showing DEGs. **(B)** Heatmap illustrating the DEGs. **(C)** Cluster dendrogram for the GSE7305 dataset. **(D)** Determination of the optimal soft threshold for the WGCNA algorithm. **(E)** Identification of co-expression modules. **(F)** Heatmap depicting module-trait correlations.

A total of 1,136 DEGs were identified in the GSE7305 dataset (disease vs. normal), comprising 572 upregulated and 564 downregulated genes (Figures 1A, B). Hierarchical clustering analysis revealed no significant outliers in the dataset, indicating that no samples needed to be excluded (Figure 1C). The optimal soft threshold, determined by selecting an *R*^2^ > 0.85 and minimal connectivity, was set at 18 (Figure 1D). This led to the identification of seven co-expression modules, with the blue module showing the strongest correlation to the disease (cor = 0.96, *p*-value < 0.05), encompassing 1,772 key module genes (Figures 1E, F).

### 3.2 Candidate genes were enriched in the NF-κB signaling pathway

**Figure 2:**
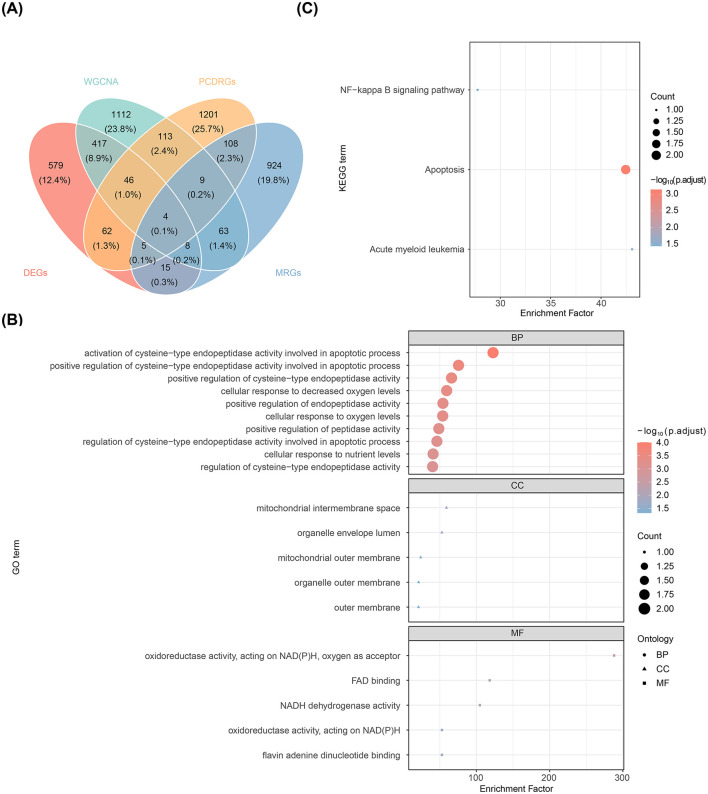
Screening and functional analysis of candidate genes. **(A)** Venn diagram displaying the overlap of genes identified through intersection analysis. **(B)** Bubble plot of enriched GO terms. **(C)** Bubble plot of enriched KEGG pathways.

Four candidate genes—FAM162A, PDK4, BCL2A1, and AIFM1—were selected by intersecting the DEGs, key module genes, MRGs, and PCD-RGs (Figure 2A). Gene Ontology (GO) enrichment analysis of these genes revealed 312 enriched terms, including 141 biological processes (BP) (e.g., activation of cysteine-type endopeptidase activity in the apoptotic process), 5 cellular components (CC) (e.g., mitochondrial intermembrane space), and 5 molecular functions (MF) (e.g., oxidoreductase activity, acting on NAD(P)H, oxygen as acceptor) (Figure 2B). Furthermore, the genes were predominantly involved in the NF-κB signaling pathway, apoptosis, and acute myeloid leukemia (Figure 2C).

### 3.3 PDK4 and AIFM1 were identified as potential biomarkers

**Figure 3:**
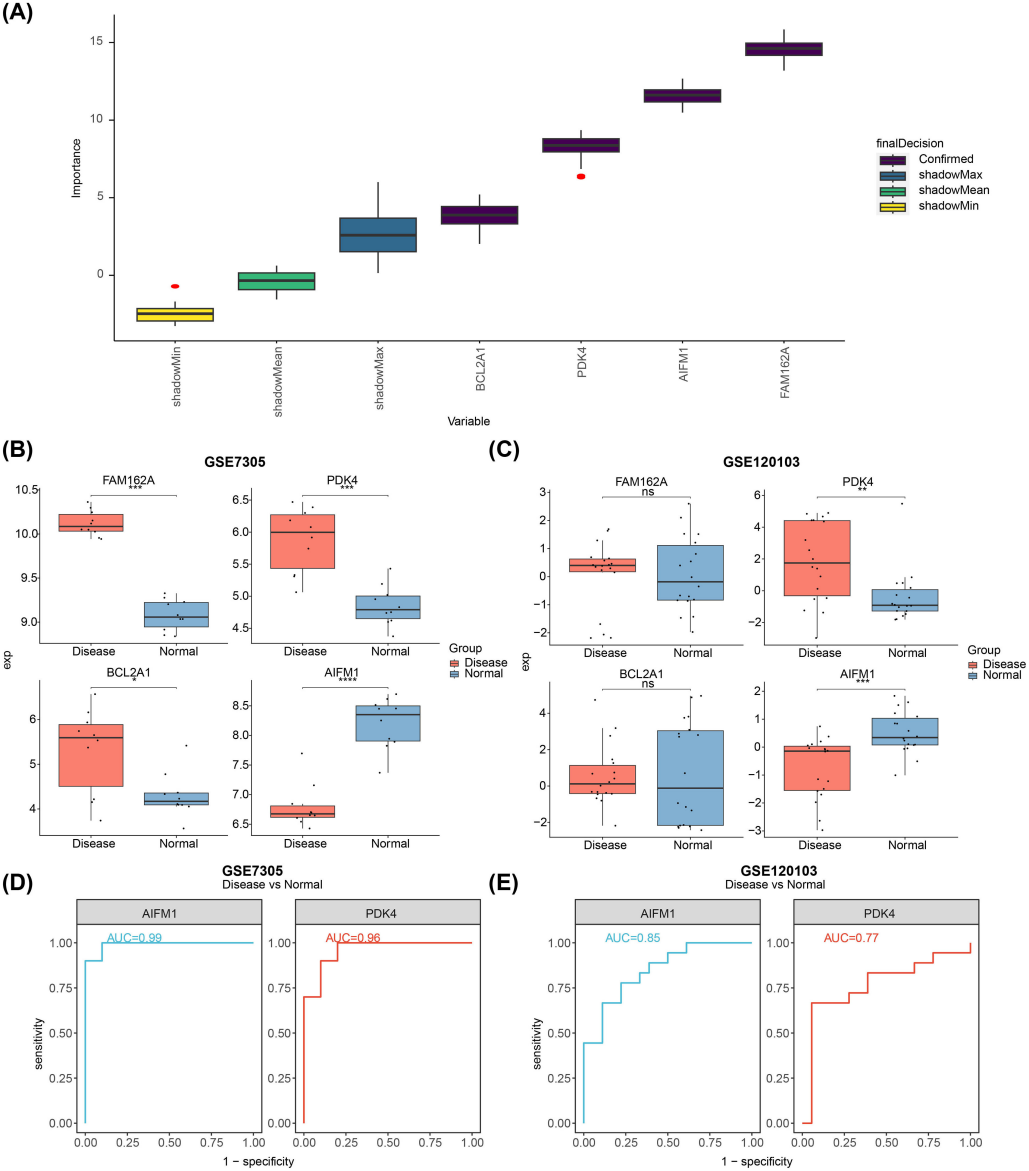
Identification of biomarkers and their differential expression in normal and diseased tissues. **(A)** Feature selection using the Boruta algorithm, where the horizontal axis represents the variable name and the vertical axis the *z*-value. **(B)** Expression levels of FAM162A, PDK4, BCL2A1, and AIFM1 in the GSE7305 dataset. **(C)** Expression levels of FAM162A, PDK4, BCL2A1, and AIFM1 in the GSE120103 dataset. **(D)** ROC curves showing AUC values for AIFM1 and PDK4 based on GSE7305. **(E)** ROC curves showing the AUC values for AIFM1 and PDK4 based on GSE120103. *p < 0.05, **p < 0.01, ***p < 0.001, ****p < 0.0001.

Feature genes (FAM162A, PDK4, BCL2A1, and AIFM1) were confirmed through Boruta analysis (Figure 3A). Expression validation in both the GSE7305 and GSE120103 datasets showed significant inter-group differences for PDK4 and AIFM1, with consistent trends across datasets. Specifically, PDK4 was upregulated in the disease group, while AIFM1 was downregulated (Figures 3B, C). The AUC values for PDK4 and AIFM1 exceeded 0.75 in both datasets, underscoring their strong diagnostic value and defining them as potential biomarkers for EMS (Figures 3D, E).

### 3.4 AIFM1 and PDK4 were enriched in the cell cycle and other pathways

**Figure 4:**
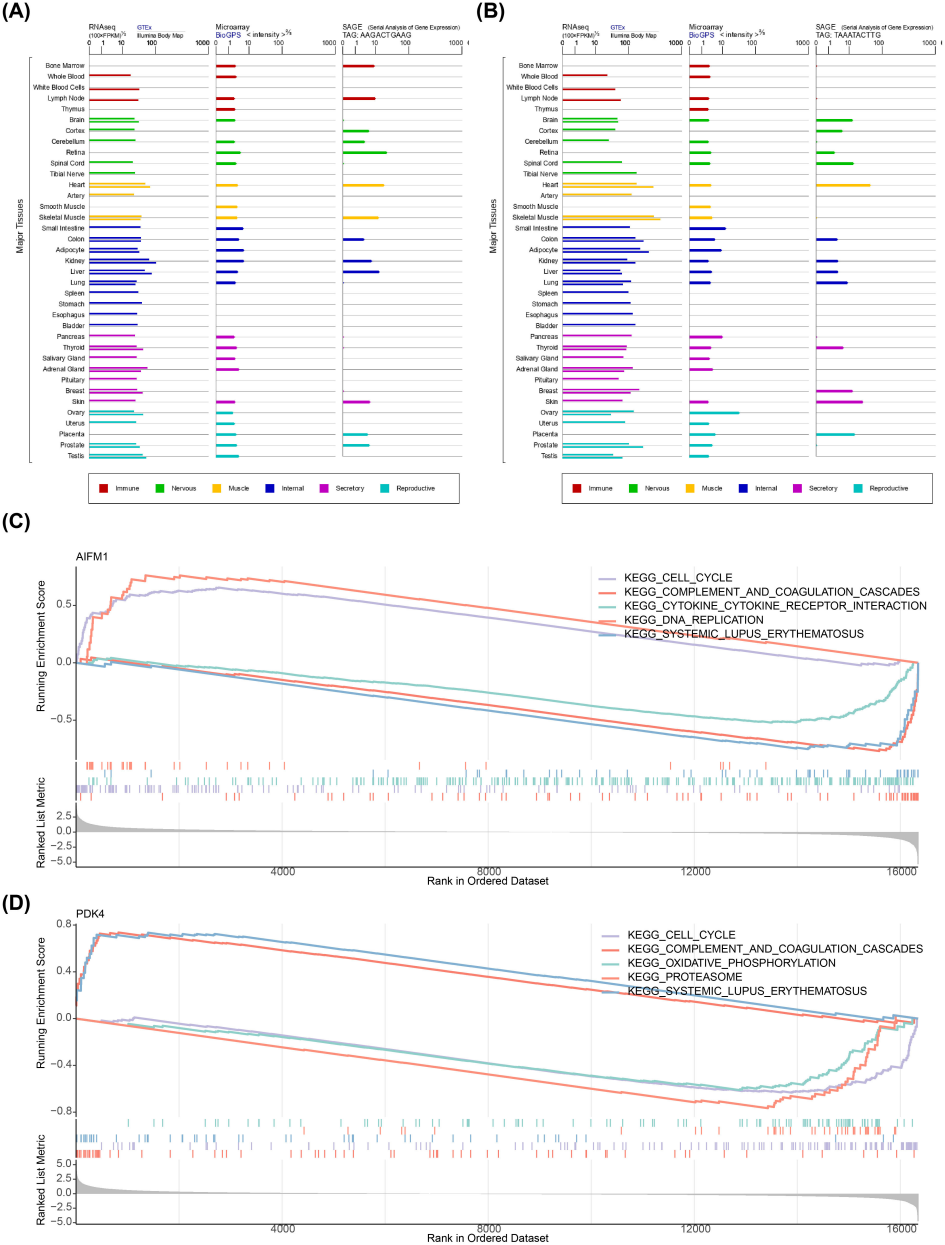
Expression of AIFM1 and PDK4 in tissues and their associated pathways. **(A)** Expression of AIFM1 across various human tissues. **(B)** Expression of PDK4 across various human tissues. **(C)** Pathway enrichment analysis of AIFM1. **(D)** Pathway enrichment analysis of PDK4.

Additionally, PDK4 exhibited higher expression in various human tissues compared to AIFM1 (Figures 4A, B). GSEA revealed that both biomarkers were significantly enriched in the cell cycle, complement and coagulation cascades, and systemic lupus erythematosus pathways (Figures 4C, D).

### 3.5 Potential biomarkers were associated with different immune cells

**Figure 5:**
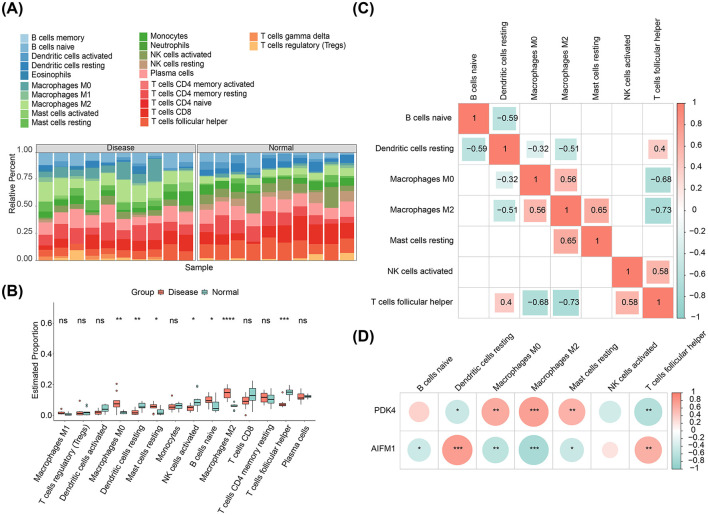
Immune infiltration analysis. **(A)** Heatmap displaying the relative abundance of 22 immune cell types in each sample from disease and control groups. **(B)** Differences in immune cell infiltration between the disease and normal groups. *p < 0.05, **p < 0.01, ***p < 0.001, ****p < 0.0001. **(C)** Correlation analysis among different immune cell types. **(D)** Correlation analysis between AIFM1, PDK4, and immune cell types.

The distribution of 22 immune infiltrating cell types in each sample from the disease and normal groups was visualized in a heatmap (Figure 5A). After excluding immune cells with an enrichment score of 0, the differences in immune cell proportions between the disease and normal groups were compared. The results revealed that M0 Macrophages, resting Mast cells, naive B cells, and M2 Macrophages were more abundant in the disease group, while resting dendritic cells, activated NK cells, and follicular helper T cells were more prevalent in the normal group (Figure 5B). Correlation analysis among the immune cell types showed the strongest positive correlation between resting mast cells and M2 Macrophages (cor = 0.65, *p*-value < 0.05), and the strongest negative correlation between follicular helper T cells and M2 Macrophages (cor = −0.73, *p*-value < 0.05; Figure 5C). Notably, resting dendritic cells showed the strongest positive correlation with AIFM1 (cor = 0.76, *p*-value < 0.001), while M2 Macrophages exhibited the strongest negative correlation with AIFM1 (cor = −0.74, *p*-value < 0.001; Figure 5D, Supplementary Tables S1, S2).

### 3.6 Two biomarkers were related to many uterine diseases

**Figure 6:**
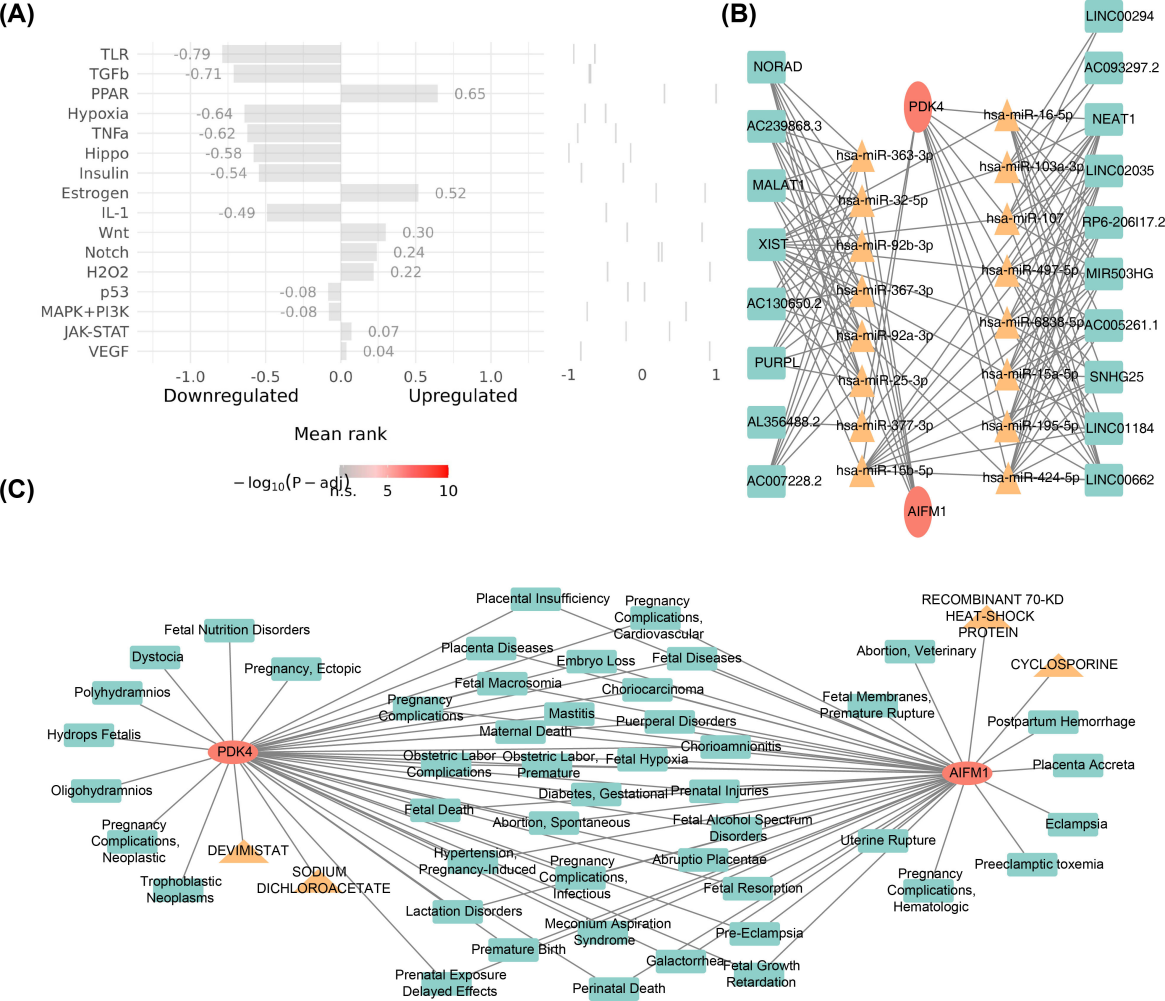
Pathways, drugs, and diseases associated with AIFM1 and PDK4. **(A)** Analysis of the upstream pathway activity of AIFM1 and PDK4. **(B)** LncRNA-miRNA-mRNA network analysis for AIFM1 and PDK4. **(C)** Identification of targeted agents for AIFM1 and PDK4, along with associated diseases.

To further investigate the upstream pathways of potential biomarkers, pathway analysis revealed that the PPAR (cor = 0.65) and Estrogen (cor = 0.52) signaling pathways exhibited the strongest activities, while the TLR (cor = −0.79) and TGFb (cor = −0.71) pathways showed the weakest activities (Figure 6A). Molecular regulatory mechanisms of potential biomarkers were further explored by constructing a lncRNA-miRNA-mRNA network. In this network, LINC00294 and XIST regulated the expression of PDK4 through hsa-miR-103a-3p, while XIST and MALAT1 regulated AIFM1 expression through hsa-miR-32-5p (Figure 6B). Additionally, four drugs were identified with potential therapeutic effects on the biomarkers: cyclosporine, recombinant 70-kd heat-shock protein, sodium dichloroacetate, and devimistat. It was also observed that both biomarkers were associated with 48 uterine diseases, including placenta accreta and hydrops fetalis (Figure 6C).

### 3.7 Single-cell data were annotated to 10 cell types

**Figure 7:**
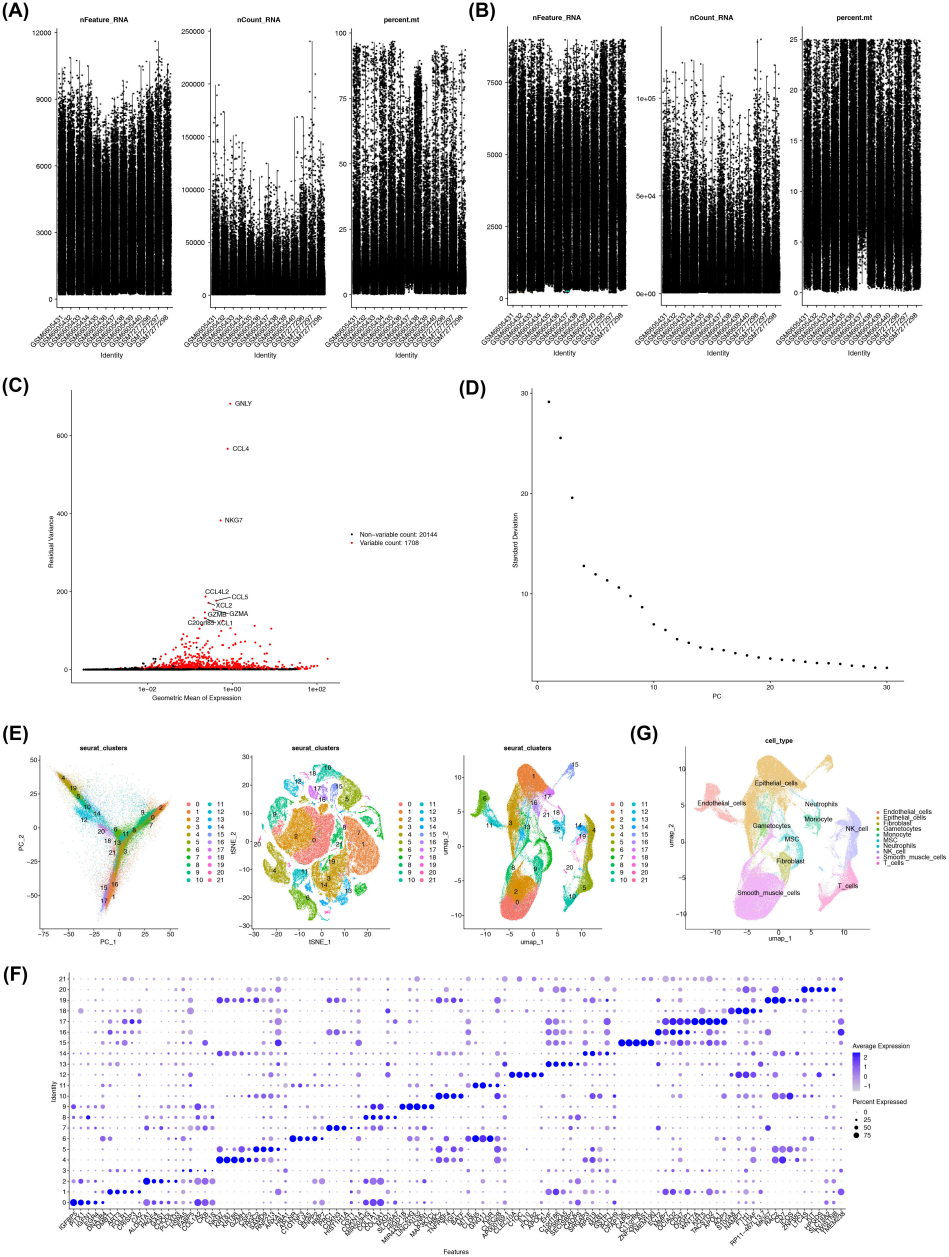
Annotation of 10 cell types associated with EMS. **(A, B)** Quality control of single-cell data from the GSE214411 dataset. Y-axis respectively stands for nFeature_RNA; nCount_RNA; percent.mt. **(C)** Identification of highly variable genes in the dataset. **(D)** Generation of elbow plots to identify PCs for subsequent analysis. **(E)** Cell clustering using the first 30 PCs, resulting in 22 distinct cell clusters. **(F)** Identification of specific highly expressed genes for each cell cluster. **(G)** Annotation of 10 cell types associated with EMS.

The GSE214411 single-cell dataset provided 26,386 genes and 139,399 cells after quality control (Figures 7A, B). From this, 2,000 highly variable genes were selected, and the top 30 PCs were used for cell clustering (Figures 7C, D). This clustering analysis identified 22 distinct cell clusters (Figure 7E), and heat maps were drawn to demonstrate the expression status of specific highly expressed genes in each cell type (Figure 7F). Further annotation of the clusters revealed 10 distinct cell types: endothelial cells, epithelial cells, gametocytes, smooth muscle cells, fibroblasts, mesenchymal stem cells (MSCs), neutrophils, monocytes, NK cells, and T cells (Figure 7G).

### 3.8 Two biomarkers showed different expression in distinct cell types between EMS and normal samples

**Figure 8:**
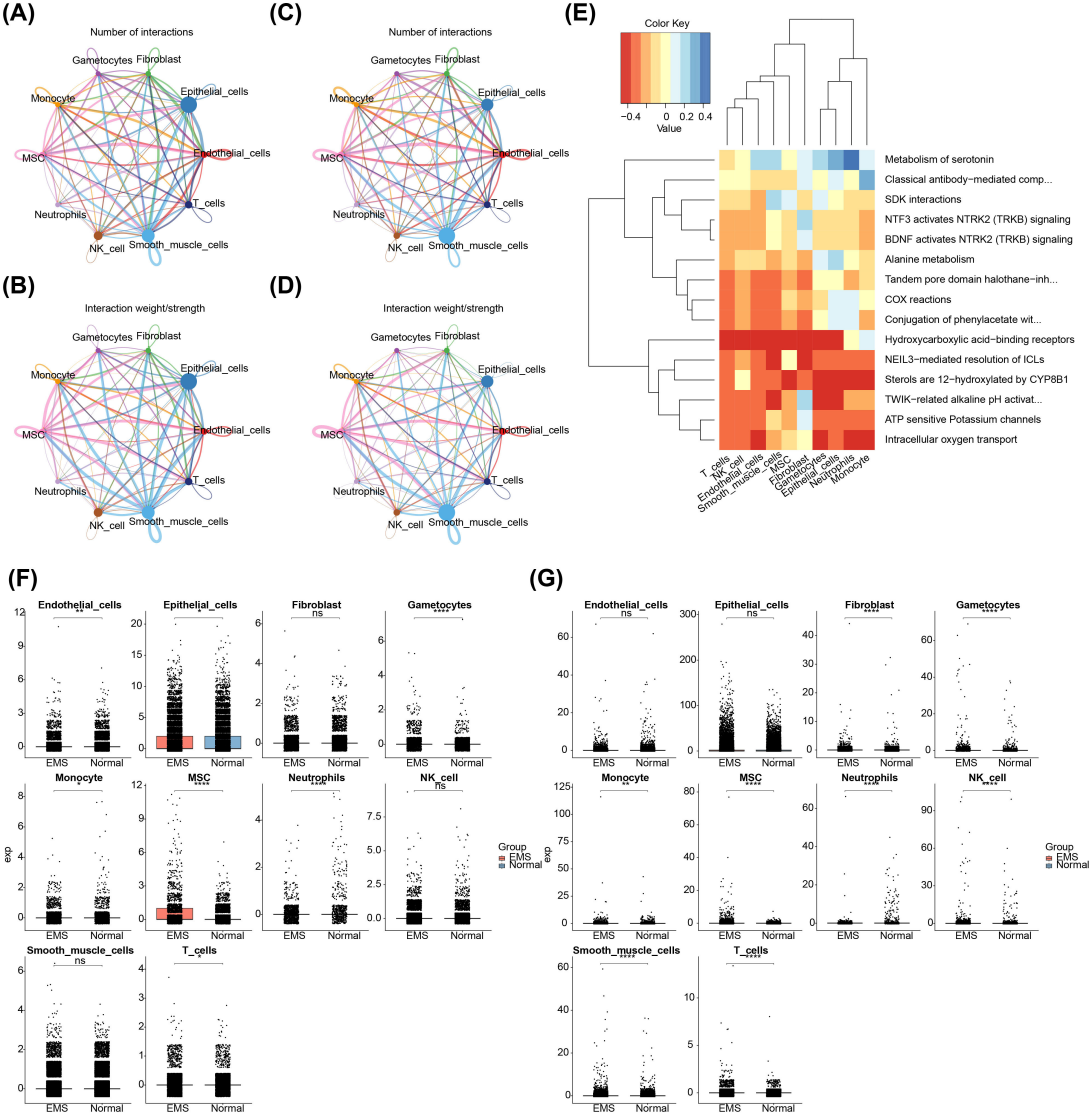
Interactions among different cell types and expression patterns of AIFM1 and PDK4 in EMS and normal samples. **(A, B)** Interaction network analysis of 10 cell types in EMS samples, highlighting the number and strength of interactions. **(C, D)** Interaction network analysis of 10 cell types in normal samples, focusing on interaction dynamics. **(E)** Pathways involved in differential expression between EMS and normal samples. **(F)** Expression levels of AIFM1 in various cell types from EMS and normal samples. **(G)** Expression levels of PDK4 in various cell types from EMS and normal samples. The y-axis stands for the normalized gene expression. *p < 0.05, **p < 0.01, ****p < 0.0001.

In the EMS samples, smooth muscle cells and endothelial cells exhibited a higher probability of interaction (Figures 8A, B). In contrast, smooth muscle cells showed a higher probability of interaction in the normal samples (Figures 8C, D). To further explore the biological significance, enrichment analysis was conducted on the differential expression between the EMS and normal samples, revealing significant enrichment in pathways related to alanine metabolism, ATP-sensitive potassium channels, and intracellular oxygen transport (Figure 8E). Differential expression analysis of AIFM1 and PDK4 between the EMS and normal groups revealed notable changes across various cell types. In the EMS group, AIFM1 was under-expressed in endothelial cells, epithelial cells, and neutrophils, while showing the opposite trend in gametocytes, MSCs, monocytes, and T cells (Figure 8F). Similarly, PDK4 in the EMS group was overexpressed in gametocytes, smooth muscle cells, fibroblasts, MSCs, monocytes, and T cells, with the opposite pattern observed in neutrophils and NK cells (Figure 8G).

### 3.9 The expression trends of AIFM1 and PDK4 were different during cell differentiation

**Figure 9:**
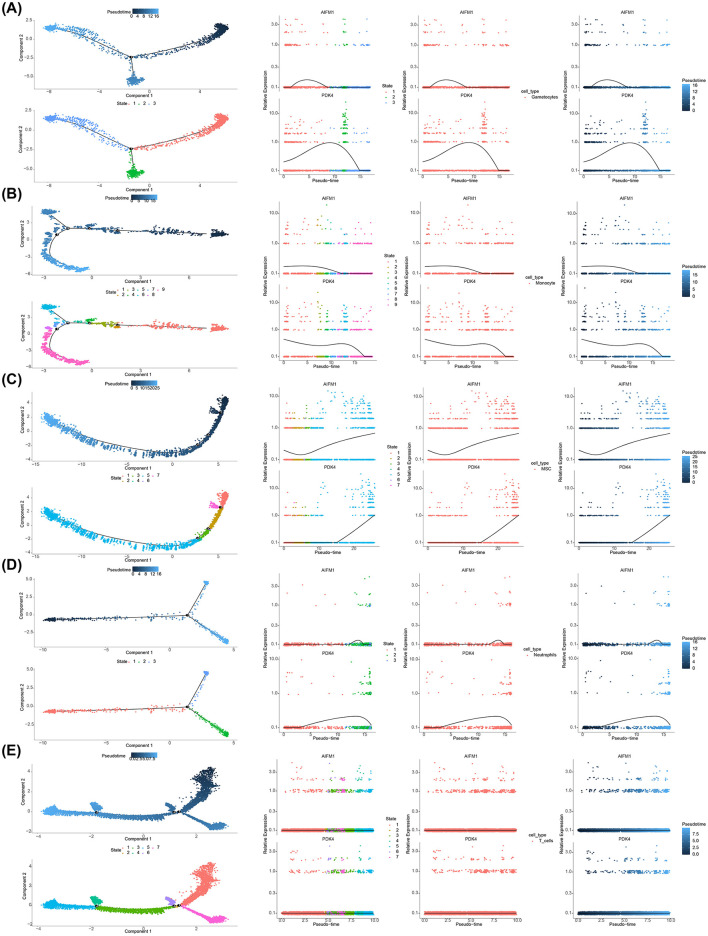
Expression trends of AIFM1 and PDK4 during cell differentiation. **(A)** Pseudotime analysis revealing expression changes of AIFM1 and PDK4 during gametocyte development. **(B)** Pseudotime analysis showing changes in the expression of AIFM1 and PDK4 during monocyte differentiation. **(C)** Pseudotime analysis of AIFM1 and PDK4 expression trends during MSC differentiation. **(D)** Pseudotime analysis illustrating the expression dynamics of AIFM1 and PDK4 during neutrophil differentiation. **(E)** Pseudotime analysis depicting the changes in AIFM1 and PDK4 expression throughout T cell differentiation.

Differentiation dynamics of these biomarkers were analyzed across various cell types. Gametocytes showed three distinct differentiation states, with AIFM1 exhibiting an initial increase followed by a decrease, while PDK4 displayed a similar trend, with an initial increase followed by a decrease throughout differentiation (Figure 9A). Monocytes had nine differentiation states, with both AIFM1 and PDK4 showing high expression in early stages that gradually decreased in later stages (Figure 9B). MSCs displayed seven differentiation states, with AIFM1 showing a decrease followed by an increase, and PDK4 exhibiting a sustained increase in the later stages (Figure 9C). Neutrophils had three differentiation states, where AIFM1 showed minimal variation, while PDK4 showed an initial increase followed by a decrease (Figure 9D). Finally, T cells had seven differentiation states, with both biomarkers showing consistently low expression levels and stable trends throughout differentiation (Figure 9E).

## 4 Discussion

EMS, a condition affecting many women of reproductive age, remains a global health challenge with an unclear pathogenesis (41). It is hypothesized that the dynamic changes in mitochondrial function, which regulate energy metabolism and redox balance, may be linked to the development of EMS (42, 43). Additionally, ferroptosis, a form of PCD, has been suggested to contribute to EMS progression through the secretion of vascular endothelial growth factors and modulation of immune mechanisms (44). While PCD is a natural cellular process, mitochondrial dysfunction can affect apoptosis and may therefore be implicated in the pathogenesis of EMS. However, the specific mechanisms linking mitochondrial dysfunction, PCD, and EMS remain to be fully elucidated, requiring further investigation.

Through a series of bioinformatics analyses—including differential expression analysis, WGCNA, machine learning, and expression validation—two potential biomarkers, AIFM1 and PDK4, were identified. Both biomarkers demonstrated strong diagnostic performance for EMS. PDK4 was upregulated, and AIFM1 was downregulated in the disease group. PDK4 encodes a mitochondrial protein that belongs to the PDK/BCKDK protein kinase family and contains a histidine kinase domain (45). It plays a pivotal role in ferroptosis resistance by inhibiting pyruvate oxidation through the pyruvate dehydrogenase pathway (46). Increased expression of PDK4 has been associated with colitis, with its substrate accumulating in CD4^+^ T cells of patients with inflammatory bowel disease. Loss of PDK4 function can delay colitis development and reduce T cell activation and aerobic glycolysis (47). Inflammation also enhances PDK4 expression in C2C12 myoblasts *via* the Jun N-terminal kinase (JNK) pathway (48). Since EMS is often accompanied by inflammation, PDK4 may mediate this pathological process through its effect on inflammatory signaling. However, the precise mechanism remains to be explored. On the other hand, AIFM1, which encodes a flavoprotein critical for nuclear breakdown during apoptosis, is located in the mitochondrial intermembrane space of healthy cells. Upon apoptosis, AIFM1 translocates to the nucleus, where it participates in chromosome condensation and fragmentation, and promotes the release of cytochrome c and caspase-9 from mitochondria (49). Previous studies have shown that silencing CCAAT/enhancer-binding protein α (C/EBPα) can promote the proliferation and anti-apoptosis of normal eutopic endometrial stromal cells, and lead to the downregulation of AIFM1 expression (50). Therefore, AIFM1 plays a pivotal role in human development and disease by serving as a key mediator of extra-mitochondrial signals.

AIFM1 and PDK4 were notably enriched in pathways related to the cell cycle, complement and coagulation cascades, as well as systemic lupus erythematosus (SLE). These pathways play pivotal roles in the pathogenesis of EMS and its associated disorders. For instance, cyclin B1 is implicated in promoting ectopic endometrial cell proliferation under ovarian hormone regulation (51). Dysregulation of cell cycle regulators interacting with p27 (Kip1) is essential in driving ovarian clear cell carcinoma progression linked to EMS, enhancing cellular proliferative activity (52). EMS is also closely associated with immune dysfunction and chronic inflammation, with the complement system playing a critical role in its pathophysiology. Studies have demonstrated the upregulation of complement components (C1S, C1QA, C1R, C3) in patients with EMS, which correlate positively with tissue factor, suggesting an interaction between the complement and coagulation pathways in EMS progression (53). Moreover, EMS and SLE, both prevalent in females, share significant immunological overlap, as SLE is a severe autoimmune condition affecting multiple systems, while EMS is a chronic inflammatory disorder involving hormonal and immune dysregulation (54, 55). Thus, AIFM1 and PDK4 may influence EMS onset and progression by modulating the cell cycle, complement and coagulation pathways, and autoimmune mechanisms.

This study underscores the pivotal role of the immune system in EMS. Immune cell infiltration levels across 22 types were analyzed, revealing a significant upregulation in M0 macrophages, resting mast cells, naive B cells, and M2 macrophages in EMS. In contrast, infiltration levels of resting dendritic cells, activated NK cells, and follicular helper T cells were downregulated. These findings suggest that the suppression of activated NK cells in endometriotic lesions may aid ectopic endometrial cells in evading immune surveillance, thereby promoting their survival and implantation. This observation aligns with previous research (56). In healthy women, peritoneal NK cells exhibit enhanced cytotoxicity; however, in patients with EMS, elevated local estrogen levels alter the number and phenotype of NK cells, impairing their ability to effectively eliminate ectopic cells and fostering lesion development (57). Correlation analysis of immune cells revealed a strong positive correlation between resting mast cells and M2 macrophages, indicating a potential synergistic role in immune regulation and inflammation resolution. Previous research has shown that the local microenvironment of EMS lesions can trigger mast cell activation, with these activated mast cells releasing various inflammatory mediators that contribute to EMS-related pain *via* inflammatory pain pathways (58). Conversely, the negative correlation between T follicular helper cells and M2 macrophages suggests a mutually restrictive relationship during immune responses. Furthermore, a strong positive correlation was observed between resting dendritic cells and AIFM1, while a negative correlation was found between M2 macrophages and AIFM1. M2 macrophages are central to the immune microenvironment in EMS (59), and blocking macrophage-associated immune checkpoint CD47 can effectively alleviate EMS (60). These findings indicate that AIFM1 may play differential regulatory roles across immune cell types, modulating the immune balance. Additionally, the expression of AIFM1 and PDK4 was correlated with immune cell infiltration levels, further suggesting that these biomarkers could serve as promising therapeutic targets for EMS.

In this study, 10 distinct cell types were identified, including endothelial cells, epithelial cells, gametocytes, smooth muscle cells, fibroblasts, MSCs, neutrophils, monocytes, NK cells, and T cells, all of which have been previously associated with EMS. Endothelial cells, for instance, play critical roles in promoting inflammation, angiogenesis, and permeability in EMS, particularly in deep infiltrating cases (61). This suggests their vital involvement in angiogenesis and the sustenance of ectopic tissues. Ectopic epithelial cells, on the other hand, resist apoptosis *via* the NNMT-FOXO1-BIM pathway and can stimulate CD4^+^ T cells through the HLA II complex, fostering chronic inflammation (62). Lymphocytes in EMS show signs of immune activation and exhaustion, with NK cells exhibiting increased cytotoxicity and T cells displaying elevated expression of checkpoint genes (63). These alterations likely contribute to the immune evasion of ectopic tissues. Fibroblasts and smooth muscle cells are also integral to pathological fibrosis and angiogenesis in EMS, leading to persistent fibrotic phenomena that contribute to classic EMS symptoms such as pain and infertility (64, 65). Monocytes and macrophages play essential roles in the progression, vascularization, and painful manifestations of EMS. Macrophages, in particular, are multifaceted cells crucial for embryonic development and tissue homeostasis in healthy conditions. However, under inflammatory stress, monocytes migrate from the bloodstream and differentiate into macrophages, which then perform functions such as infection defense and wound healing. The role of macrophages in inflammation has garnered significant attention in recent research (66). Neutrophil levels are notably elevated in the blood, peritoneal fluid, and ectopic endometrium of patients with EMS. Ectopic endometrial lesions actively recruit neutrophils, which, in turn, promote early-stage angiogenesis within the inflammatory microenvironment, thereby creating a positive feedback loop (67, 68).

In EMS samples, smooth muscle cells and endothelial cells exhibited a higher likelihood of interaction, suggesting abnormal intercellular communication and signaling in the pathological context of EMS. Such dysregulated interactions may contribute to inflammation, tissue adhesion, and lesion formation in EMS. Consequently, targeting the crosstalk between these two cell types could offer a novel therapeutic approach for EMS treatment. The distinct expression patterns of AIFM1 and PDK4 in various cell types highlight their specific roles in the disease, underscoring their potential as biomarkers for understanding the immune microenvironment of EMS. This understanding is crucial for developing more targeted and precise treatment strategies.

## 5 Conclusion

This study identified potential biomarkers, AIFM1 and PDK4, in the pathogenesis of EMS through comprehensive bioinformatics analysis. These biomarkers effectively distinguished EMS from healthy controls. Additionally, by examining the communication between cells and the signaling pathways involved, this research provides valuable insights for diagnosing and treating EMS. However, the study has some limitations, including the absence of experimental validation of the biomarkers' functional roles and a relatively small sample size. Future experimental studies are needed to validate the conclusions derived from this bioinformatics analysis.

## Data Availability

The original contributions presented in the study are publicly available. This data can be found here: https://www.ncbi.nlm.nih.gov/geo with accession numbers GSE7305, GSE120103, GSE214411 and also the MitoCarta 3.0 database http://www.broadinstitute.org/mitocarta. Further inquiries can directed to the corresponding author.
